# SOCIAL CONTEXTS OF FINANCIAL EXPLOITATION OF OLDER ADULTS IN MONGOLIA: AN EXPLORATORY STUDY

**DOI:** 10.1093/geroni/igad104.2509

**Published:** 2023-12-21

**Authors:** Ariunsanaa Bagaajav

**Affiliations:** City University of New York, New York City, New York, United States

## Abstract

Financial exploitation (FE) is a type of elder abuse and is the illegal or improper use of funds and resources of older persons. Approximately one in 15 older adults are victimized by FE globally. Due to its devastating consequences, FE has been identified as an emerging health, financial, and social crisis. Evidence suggests exploring cultural values is crucial to understanding the nature of FE in a specific context. While conceptualization of FE varies significantly across cultures, little is known about FE in Mongolia given its potential for increased vulnerability. We present findings of a secondary analysis of previously conducted exploratory study in Ulaanbaatar, Mongolia. A total of 24 physicians participated in semi-structured interviews, participants who were uniquely positioned to encounter incidences of FE due to requent contact with older patients. All interviews were audiotaped and transcribed verbatim. Grounded theory methods were used for the data analysis. This study found that older Mongolians are at increased vulnerability to financial exploitation due to mounting financial hardships their families experience. To assist, older adults take out pension loans with high interest fees. Due to a lack of personal fund, essential needs of older adults are often ignored. In some instances, older adults’ pensions could be the most reliable source of income for poor families. Physicians believe while some older adults act upon willingly to assist, some might be forced under the collective decision made by family members. The findings of this study advanced the knowledge of FE in Mongolia which was formerly understudied.

